# Supplementary material for: From sequence to enzyme mechanism using multi-label machine learning
Source: BMC Bioinformatics. 2014 May 19;15:150. doi: 10.1186/1471-2105-15-150 (PMC4229970; doi:10.1186/1471-2105-15-150)
Supplement: Additional file 2 — Java code of ml2db. Additional file ml2db_code.tar.gz contains the Java source code to run the multi-label machine learning experiments and save the results to database. The code’s Javadoc is included. [file 1471-2105-15-150-S2.zip › additional file 2/ml2db/ml2db/doc/mulan/transformations/multiclass/SelectBasedOnFrequency.html]

SelectBasedOnFrequency


JavaScript is disabled on your browser.


- Overview
- Package
- Class
- Use
- Tree
- Deprecated
- Index
- Help

- Prev Class
- Next Class

- Frames
- No Frames

- All Classes

- Summary:
- Nested |
- Field |
- Constr |
- Method

- Detail:
- Field |
- Constr |
- Method


mulan.transformations.multiclass

## Class SelectBasedOnFrequency

- java.lang.Object
- - mulan.transformations.multiclass.MultiClassTransformationBase
  - - mulan.transformations.multiclass.SelectBasedOnFrequency

- All Implemented Interfaces:
  :   java.io.Serializable, MultiClassTransformation

  ---

    

  ```
  public class SelectBasedOnFrequency
  extends MultiClassTransformationBase
  ```

  Class that implement the Select-Max and Select-Min transformation methods.

  Version:
  :   2012.02.02

  Author:
  :   Stavros Bakirtzoglou

  See Also:
  :   Serialized Form

- - ### Constructor Summary

    Constructors

    Constructor and Description

    `SelectBasedOnFrequency(SelectionType type)`

    Initializes the transformation with a `SelectionType`
  - ### Method Summary

    Methods

    | Modifier and Type | Method and Description |
    | --- | --- |
    | `weka.core.Instances` | `transformInstances(MultiLabelInstances mlData)` Transforms a multi-label dataset to a multi-class single label dataset |

    - ### Methods inherited from class java.lang.Object

      `equals, getClass, hashCode, notify, notifyAll, toString, wait, wait, wait`

- - ### Constructor Detail


    - #### SelectBasedOnFrequency

      ```
      public SelectBasedOnFrequency(SelectionType type)
      ```

      Initializes the transformation with a `SelectionType`

      Parameters:
      :   `type` - type of frequency-based selection (MIN/MAX)
  - ### Method Detail


    - #### transformInstances

      ```
      public weka.core.Instances transformInstances(MultiLabelInstances mlData)
                                             throws java.lang.Exception
      ```

      **Description copied from interface: `MultiClassTransformation`**

      Transforms a multi-label dataset to a multi-class single label dataset

      **Specified by:**
      :   `transformInstances` in interface `MultiClassTransformation`

      **Overrides:**
      :   `transformInstances` in class `MultiClassTransformationBase`

      Parameters:
      :   `mlData` - a multi-label dataset

      Returns:
      :   a single-label multi-class dataset

      Throws:
      :   `java.lang.Exception`


- Overview
- Package
- Class
- Use
- Tree
- Deprecated
- Index
- Help

- Prev Class
- Next Class

- Frames
- No Frames

- All Classes

- Summary:
- Nested |
- Field |
- Constr |
- Method

- Detail:
- Field |
- Constr |
- Method
